# Investigating Reading Comprehension in Adolescents with Intellectual Disabilities: Evaluating the Simple View of Reading

**DOI:** 10.5334/joc.188

**Published:** 2021-09-13

**Authors:** Karin Nilsson, Henrik Danielsson, Åsa Elwér, David Messer, Lucy Henry, Stefan Samuelsson

**Affiliations:** 1 Linköping University, Sweden; 2 The Swedish Institute for Disability Research, Sweden; 3 The Open University, UK; 4 City, University of London, UK

**Keywords:** reading comprehension, intellectual disability, simple view of reading, vocabulary, delay, difference, structural equation modelling

## Abstract

Reading comprehension difficulties are common in individuals with intellectual disabilities (ID), but the influences of underlying abilities related to reading comprehension in this group have rarely been investigated. One aim of this study was to investigate the Simple View of Reading as a theoretical framework to describe cognitive and linguistic abilities predicting individual differences in reading comprehension in adolescents with non-specific ID. A second aim was to investigate whether predictors of listening comprehension and reading comprehension suggest that individuals with ID have a delayed pattern of development (copying early grade variance in reading comprehension) or a different pattern of development involving a new or an unusual pattern of cognitive and linguistic predictors. A sample of 136 adolescents with non-specific ID was assessed on reading comprehension, decoding, linguistic, and cognitive measures. The hypotheses were evaluated using structural equation models. The results showed that the Simple View of Reading was not applicable in explaining reading comprehension in this group, however, the concurrent predictors of comprehension (vocabulary and phonological executive-loaded working memory) followed a delayed profile.

## Introduction

Intellectual disability (ID) involves general cognitive impairments that concern conceptual, social, and adaptive abilities, with standardized scores on tests of IQ below 70 (American Psychiatric Association, 2013). Students with ID have delays in a range of cognitive and language abilities (Danielsson, Henry, Messer, & Rönnberg, 2012; Danielsson, Henry, Rönnberg, & Nilsson, 2010; Henry, 2001; Henry & Winfield, 2010; Molen, Henry, & Luit, 2014) including reading comprehension. Jones, Long, and Finlay (2006) found that adults with ID exhibited a ‘reading-comprehension-age’ between 6:0–9:6 years. It also appears that in a comparison of different disability groups, children with ID have the lowest performance on reading comprehension assessments (Wei, Blackorby, & Schiller, 2011). ID is defined in part by IQ (American Psychiatric Association, 2013) but reading comprehension reflects much more than IQ. It is a multifaceted process that begins with processes such as the fine coordination of eye movements and ends with the processing of semantic information (Kintsch & Rawson, 2005; Rayner, Juhasz, & Pollatsek, 2005). The literature on reading comprehension in ID is sparse, with the result that explanations for their reading comprehension problems are largely absent.

The aim of the present study was to investigate the pattern of contributing variables in relation to reading comprehension in students with non-specific ID. More specifically, the applicability of the ‘Simple View of Reading’ (Gough & Tunmer, 1986; Hoover & Gough, 1990) was tested. Further, close attention was paid to identifying how cognition, language, and home literacy contributed to reading comprehension in this group. This investigation also involved testing whether the cognitive and language profiles of students with ID were compatible with a delayed development profile or a different profile, compared to typical readers. Even if the Simple View of Reading is one of the leading theories in reading research, there are other theories emphasizing additional variables. The Lexical Quality Hypothesis (Perfetti, 2007) states that variation in the quality of word representations has consequences for reading comprehension, meaning that vocabulary could play a key role in reading comprehension. The Reading Systems Framework (Perfetti & Stafura, 2014) is a general, and broader, framework that takes more components of reading comprehension into consideration while emphasising word knowledge as a key process. Furthermore, Verhoeven and van Leeuwe (2008) proposed that a combination of Simple View of Reading and the Lexical Quality Hypothesis provided the best fit for explaining reading comprehension in typically developing children. However, because research on reading comprehension and its predictors in individuals with ID is in its infancy, the present study focused on evaluating the applicability of one of the leading theories, namely the Simple View of Reading.

### The Simple View of Reading

According to the Simple View of Reading (SVR), decoding and listening comprehension (i.e. of spoken language) provide the basis for explaining reading comprehension. The SVR assumes that Reading (R) is a product of Decoding (D) and Comprehension (C), thus, R = D × C (Gough & Tunmer, 1986). There is a large body of research supporting the Simple View of Reading in typically developing children (e.g. Garcia & Cain, 2014; Torppa et al., 2016). For example, a longitudinal study by Lervåg, Hulme, and Melby-Lervåg (2017) showed that 96% of the variation in reading comprehension skills was explained by decoding and listening comprehension in Norwegian speaking children. Different approaches have been used when assessing the components in the SVR, and composites of different measures are commonly chosen (LARRC, 2017). In the present study we used a composite of timed and untimed word and nonword reading tasks to assess the decoding component, and listening comprehension as the proxy for language comprehension as stated in the original article by Gough and Tunmer (1986). All other measures in this study (i.e. grammatical understanding, vocabulary, phonological executive-loaded working memory, phonological short-term memory, mental age, verbal fluency, rapid automatized naming, phonological awareness, visual short-term memory, visuospatial executive-loaded working memory, spatial short-term memory, and home literacy) were used as predictors, and will henceforth be named predictors. The following assumptions from the SVR were examined in this study. First, all included predictors are mediated through the two components, decoding and listening comprehension, and none of the included predictors make individual direct contributions to reading comprehension. Second, the combination of the two components, expressed as a product, is the best description of reading comprehension. The SVR indicates that reading comprehension difficulties can be due to impaired decoding, impaired listening comprehension, or both (Gough & Tunmer, 1986). Several studies have found that across age decoding decreases in importance, as it is mastered, while listening comprehension increases in importance (Juel, Griffith, & Gough, 1986; Lervåg, Hulme, & Melby-Lervåg, 2017).

In general terms, there is also support for the SVR from studies of children with reading difficulties such as dyslexia, showing that their difficulties with decoding affect reading comprehension because the children fail to access the text (Catts, Kamhi, & Adlof, 2012). There is also support for the SVR from studies on children with comprehension difficulties. One study showed that around 10% of primary school children who had adequate or even exceptionally good decoding abilities, exhibited much poorer abilities in reading comprehension (Hulme & Snowling, 2011). These individuals are often referred to as ‘poor comprehenders’ (Nation, 2005), and their difficulties with reading comprehension are due to compromised listening comprehension (Nation, Cocksey, Taylor, & Bishop, 2010). Further, a combination of difficulties in decoding and listening comprehension, results in compromised reading comprehension. These individuals can have severe reading comprehension deficits, and are often referred to as ‘garden-variety poor readers’ (Stanovich, 1988).

A limited number of investigations have examined the SVR in students with ID. One study found that decoding and listening comprehension were significant predictors of reading comprehension for explicit content (i.e. lower level reading comprehension) in students with mixed-aetiology ID, after controlling for nonverbal reasoning. However, the only significant predictor for implicit content (i.e. higher level reading comprehension) was nonverbal reasoning (van Wingerden, Segers, van Balkom, & Verhoeven, 2014). These results suggest that the SVR is only applicable for reading comprehension of explicit content. In addition, other studies on students with mixed-aetiology ID have found that decoding and listening comprehension play a crucial role in reading comprehension (van Wingerden, Segers, & van Balkom, 2017; Verhoeven & Vermeer, 2006), hence, lending support to the SVR.

In summary, the SVR has widespread acceptance and supporting evidence from a number of investigations. There are, however, studies that have found patterns inconsistent with the SVR such that measures of decoding and listening comprehension are insufficient to account for reading comprehension performance (Kirby & Savage, 2008; Ouellette & Beers, 2010). In these studies, it is suggested that additional abilities have a direct impact on reading comprehension.

### Predictors contributing to comprehension

While the investigation of the SVR provides an explanation of the structure of reading comprehension in students with non-specific ID, the current study also involves an evaluation of delay and difference hypotheses with regard to reading and listening comprehension. A delay hypothesis would suggest that the predictor variables contributing to comprehension in adolescents with ID will resemble the predictors found in previous research on younger typically developing children (although the end point in development may be at a lower level for those with ID). A difference hypothesis would suggest that the predictors contributing to comprehension in adolescents with ID will be different from the predictors found in previous research on typically developing students. Patterns consistent with each of these hypotheses will be described below. As comprehension is a complex process, requiring simultaneous processing of key information and logical reasoning/inference, it is not surprising that a number of different predictors may contribute to explaining the variance in both reading and listening comprehension. Beyond the predictors associated with the SVR presented above, reading and listening comprehension in typically developing students are mainly explained by IQ, vocabulary, phonological executive-loaded working memory (ELWM), phonological short-term memory, and grammatical skills (Braze, Tabor, Shankweiler, & Mencl, 2007; Cain, Oakhill, & Bryant, 2004; Christopher et al., 2012; Hulslander, Olson, Willcutt, & Wadsworth, 2010; Kim, 2015, 2016; Muter, Hulme, Snowling, & Stevenson, 2004; Ouellette, 2006; Ouellette & Beers, 2010; Segers, Damhuis, Sande, & Verhoeven, 2016; Tiu, Thompson, & Lewis, 2003). In addition to the main predictors which are consistently found in previous research other predictors, such as semantic fluency, rapid automatized naming (RAN) and home environment have been suggested to influence reading comprehension. One study found that semantic fluency, whereby participants were asked to verbalize as many names of animals as possible within 60 seconds, accounted for additional variance in reading comprehension over and above vocabulary size (Nouwens, Groen, Kleemans, & Verhoeven, 2017). Another study found that a measure of RAN obtained in kindergarten, was significantly related to later measures of passage comprehension (Parrila, Kirby, & McQuarrie, 2004). The role of environmental factors is often investigated in research on reading abilities. For example, Segers, Damhuis, Sande, and Verhoeven (2016) reported that home environment factors (i.e. reading frequency, reading climate, and parent education) were related to reading comprehension via phonological awareness, word decoding, and vocabulary. Another study showed that parent literacy accounted for a significant part of the variance in reading comprehension in a group of children with reading disabilities (Rashid, Morris, & Sevcik, 2005).

Studies examining the predictive role of IQ in reading comprehension show very different results. The role played by IQ in identifying children with reading disabilities, as well as these children’s potential to take advantage of instruction, has been challenged (Fletcher et al., 1994; Stanovich, Cunningham, & Feeman, 1984; Vellutino, Scanlon, & Lyon, 2000). A common standpoint is that IQ is less useful compared to language based skills in identifying disability groups and predicting groups’ potential of growth. However, in a review, Fuchs and Young (2006) concluded that IQ was a unique significant predictor of gains in reading, this was particularly the case for reading comprehension. Their studies included students up to grade 5 who were involved in reading remediation programs. In older students, studies suggest both no predictive impact of full scale IQ on reading comprehension (Scarborough, 1998) and significant predictive impact (Hulslander, Olson, Willcutt, & Wadsworth, 2010). In poor comprehenders, deficits in verbal abilities are well established (Elwér, Keenan, Olson, Byrne, & Samuelsson, 2013; Nation, Cocksey, Taylor, & Bishop, 2010). Deficits in non-verbal ability are commonly not reported (Nation & Snowling, 1998; Stothard & Hulme, 1992). Although, Nation, Clarke, and Snowling (2002) found that a subgroup of their poor comprehenders had compromised non-verbal abilities compared to typical readers. Interestingly, this subgroup of readers did not show more severe reading comprehension difficulties compared to poor comprehenders with adequate non-verbal ability. Thus, the results concerning the relationship between IQ and reading comprehension are inconclusive and the effect of IQ in groups of students with ID is still an open question.

In the few studies that have examined reading comprehension in students with ID, support for both the delay and difference hypotheses has been found. In studies of both typically developing students and of students with ID, nonverbal IQ has been found to predict reading comprehension directly (van Wingerden, Segers, & van Balkom, 2017; van Wingerden, Segers, van Balkom, & Verhoeven, 2018; Verhoeven & Vermeer, 2006), supporting a delay hypothesis as the same relations are found in both groups of students. Other studies have found different patterns of reading comprehension predictors in students with ID when compared to those with typical development. In one study, early literacy skills (a composite variable calculated from measures of phonological awareness and letter knowledge) had a direct and strong relationship to reading comprehension, over and above decoding and language comprehension, in students with mixed-aetiology ID (van Wingerden, Segers, & van Balkom, 2017). The same research team conducted a follow-up study, presenting longitudinal data, where early literacy skills continued to predict reading comprehension in addition to prior reading comprehension, decoding, listening comprehension, and nonverbal reasoning (van Wingerden, Segers, van Balkom, & Verhoeven, 2018). In addition, a two-year longitudinal study of students with non-specific ID found that phonological awareness and letter-sound knowledge were significantly related to improvement in reading comprehension together with age and type of school placement (Sermier Dessemontet & Chambrier, 2015). These results differ from findings in typically developing children, where phonological awareness explains decoding abilities rather than reading comprehension (Oakhill & Cain, 2012). In summary, previous studies suggest that reading and listening comprehension rely on both cognitive and linguistic skills. For typically developing children, the most important skills were IQ, vocabulary, phonological ELWM, and grammatical skills whereas for students with ID the most important skills were IQ, phonological awareness, and letter-sound knowledge.

### The present study

In the present study, 136 participants with non-specific ID between 12–19 years of age were assessed on reading comprehension, listening comprehension, decoding, a range of cognitive and language variables, and home literacy. All variables included in our study were chosen on the basis that they have correlated with reading abilities in previous research. We chose not to include the variable letter-sound knowledge, because that measure is more reliable as a longitudinal predictor when assessed before children have started their formal reading instruction or in the early school years (Sermier Dessemontet & Chambrier, 2015; van Tilborg, Segers, van Balkom, & Verhoeven, 2014). In another article on the same data set, our research team have outlined the variables contributing to decoding skills in adolescents with ID (Nilsson et al., 2021). Therefore, the aim of the present study was to investigate the pattern of contributing variables in relation to reading comprehension in students with non-specific ID.

### Research questions and hypotheses

Very little is known about reading comprehension in adolescents with ID. As the literature is scarce we validated the most common framework of reading, the Simple View of Reading in this group of readers. Previous studies have found low levels of reading comprehension in adolescents with ID. In this study we aimed to determine whether the Simple View of Reading could explain reading comprehension in adolescents with ID, and whether the pattern of prediction corresponded with that found in younger typically developing children. Two research questions with alternative hypotheses were evaluated with Structural Equation Modelling (SEM).

**Figure 1:**
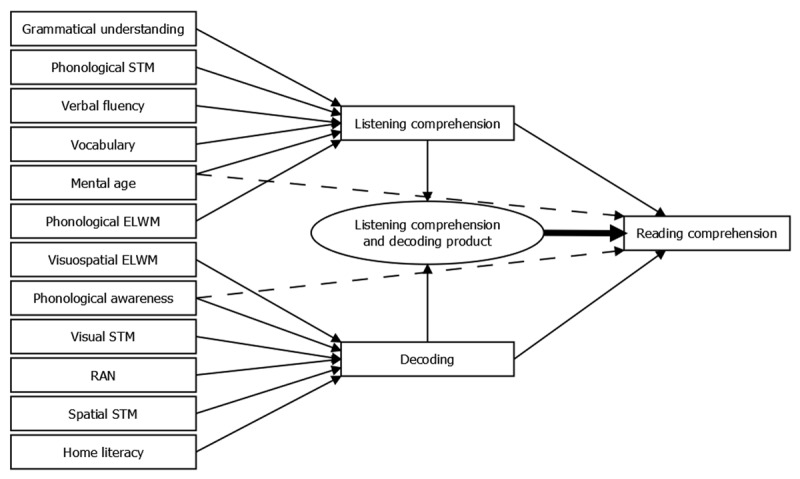
Diagram of the Simple View of Reading hypotheses.

**Figure 2:**
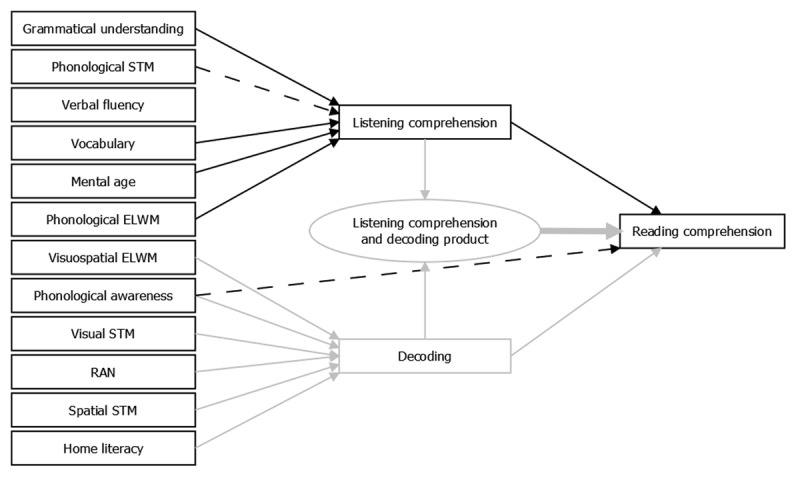
Diagram of the delay and difference hypotheses.

1. Is the simple view of reading applicable when explaining reading comprehension in adolescents with ID?
  Hypothesis 1a, the Simple view of Reading hypothesis, is applicable if the following two patterns of results are supported in the best model of the data. First, the product of decoding and listening comprehension influences reading comprehension (thick line in ***Figure 1***). Second, all other included variables are fully mediated through listening comprehension or decoding (no direct connections to reading comprehension). Otherwise, the SVR is not applicable (Hypothesis 1b). In ***Figure 1***, two direct, unmediated connections between our predictor variables and reading comprehension are shown by dashed lines, such connections could occur between any predictor variables and reading comprehension, only two are included in the ***Figure 1*** for illustrative purposes. If any dashed lines are included in the best model, hypothesis 1b will be supported.
2. Do the variables contributing to reading and listening comprehension in adolescents with ID follow a delay or a difference model?
  Hypothesis 2a, the delay hypothesis, is accepted if the best model of reading comprehension contains the same predicting variables of reading and listening comprehension skills as in previous literature on younger typically developing children (i.e. grammatical skills, vocabulary, mental age, and phonological executive-loaded working memory). In ***Figure 2***, these connections are indicated by the black solid lines. Hypothesis 2b, the difference hypothesis, is accepted if the best model of reading comprehension has different predicting variables of reading and listening comprehension skills compared to younger typically developing children (e.g. a direct connection involving phonological awareness, as in Sermier Dessemontet & Chambrier, 2015; van Wingerden, Segers, & van Balkom, 2017). In other words, this hypothesis is considered as applicable if the best fitting model contains a connection between the predictor variables and reading and listening comprehension which differs from the ones outlined in Hypothesis 2a. In ***Figure 2***, this is illustrated by the presence of two dashed lines, although the direct connections could occur between any of the predictor variables and reading and listening comprehension.

## Method

The data collection for this study is part of a larger project on reading ability in students with ID. More detailed descriptions of the method can be found in Nilsson et al. (2021).

### Participants

To be able to conduct analyses that examine the fit of the SVR, and the relationship between predictor variables and comprehension in students with non-specific ID, relevant data was collected from a large sample of 136 adolescents with ID. In the area of structural equation modelling, where path analysis belongs, it is complicated to estimate the required sample size. Common guidelines described in Wolf, Harrington, Clark, and Miller (2015) are: 1) a minimal sample size of 100–200 participants depending on recommendation chosen (e.g., 150 in Anderson and Gerbing (1988)); 2) a minimum of 5 (Bentler & Chou, 1987) to 10 (Bollen, 1989) participants per estimated parameter; and 3) 10 cases per variable. The current study had 15 variables and 31 parameters to estimate in the initial model with all predictor variables allowed to affect both decoding and listening comprehension. The predicted models had about 15 parameters to estimate (covariance between predictor variables were allowed but it was not certain how many would be included, therefore a precise number was hard to determine before the model was evaluated). Given these guidelines (10 participants per 15 variables = 150; 10 participants per 15 estimated parameters = 150), it is reasonable to have a sample size of 150 participants. Recent simulation studies (Muthén & Muthén, 2009; Wolf, Harrington, Clark, & Miller, 2015) have shown that required sample size also depends on many other factors, including distribution of the variables, amount of missing data, reliability of the variables, and strength of the relations among the variables. For a confirmatory factor analysis, simulations showed that with normally distributed variables and no missing data, 150 participants gave a power of 0.81 (Muthén & Muthén, 2009). For a path analysis simulation it was found that under ideal conditions, a minimum sample size of 70 would be needed to detect an indirect effect with a power of 0.81 (Wolf, Harrington, Clark, & Miller, 2015). The reliability of the measures was particularly important in path analysis and it was advised to include measurement errors in the model to increase power, which we did. It is anticipated that the proposed study will have variables with normal distributions, low amounts of missing data, high reliability of tests (for the tests that have reliability estimates), and relatively few parameters to estimate in the final model. Taken together, 150 participants were considered a reasonable sample size.

The planned inclusion criteria were: 1) age 12–19 years; 2) decoding ability that could be measured with the tests used in this study (i.e. >0 words read correctly); 3) normal or corrected to normal vision and hearing; 4) Swedish speaking home environment since birth; and 5) no developmental diagnoses other than non-specific ID. Because comorbidities with other diagnoses, such as ADHD and ASD, are common in individuals with ID, if the recruitment of participants proved too challenging (less than 50 participants recruited in 6 months of total recruitment/testing time), the fifth inclusion criterion was to be dropped. However, due to financial constraints, data collection was planned to be stopped after two years or 150 included participants, whichever happened first. After 6 months, only 17 participants were recruited. Hence, the fifth inclusion criterion was dropped, which meant that 51% of the included participants were reported to have additional diagnoses. After two years, the data collection ended with 136 participants tested. However, the target of 150 participants would have been reached if the pandemic had not impacted testing of participants with consent the final 2 months of data collection. Participants were recruited via schools (upper secondary and high school) in Sweden. After initial contact from the research team, principals or teachers contacted students and parents. To be included in the study everyone involved (i.e. schools, parents, and adolescents) had to provide their consent. Participants and parents were initially asked to sign a letter of consent but all participants were also asked for oral assent before the assessment started.

We received a total of 176 consent letters, and 15 were excluded before testing due to the following reasons: presence of a syndrome (3); not speaking Swedish in home environment (6); not correct chronological age (2); and no name or contact information was included (4). In addition, 22 participants were not tested due to pandemic related school restrictions. Of the 139 tested participants, one was excluded because of inclusion criterion two (decoding was not tested), and two were excluded because of inclusion criterion three (not normal or corrected to normal hearing or hearing not tested). Our final sample consisted of 136 participants (59 girls). This sample size was considered large enough to proceed with our planned analysis. The mean chronological age was 189.61 months (*SD* = 25.87 months), the mean estimated IQ level of the participants was 59.43 (*SD* = 9.72), and the mean mental age was 112.88 months (*SD* = 25.26 months). More detailed information is provided in ***Table 1***. IQ level was estimated using two sub-tests from the WISC-V (Wechsler, 2014). Fifteen participants were estimated to have an IQ above 70, however, all participants were enrolled in special education classes during the data collection, which in Sweden means that they have been thoroughly tested and diagnosed as having ID and an IQ < 70 by a clinician.

**Table 1:** Descriptive statistics of participant characteristics and task performances (raw scores) of adolescents with intellectual disability (n = 136).


TEST	*M*	*SD*	MIN	MAX	SKEWNESS	KURTOSIS

Chronological age (months)	189.61	25.87	146	239	0.26	–1.02

Mental age (months)	112.88	25.26	63	190	0.62	0.31

IQ	59.43	9.72	40	88	0.30	–0.08

Reading comprehension	18.04	12.54	2	56	0.81	0.10

Listening comprehension	9.28	3.73	0	15	–0.57	–0.17

Word recognition timed	45.10	17.76	4	94	0.03	–0.55

Word recognition untimed	76.48	18.87	13	99	–1.02	0.32

Phonological decoding timed	23.28	11.75	2	55	0.32	–0.71

Phonological decoding untimed	36.18	16.38	2	61	–0.40	–1.02

Blending	15.71	3.66	2	20	–0.94	0.47

Elision	9.91	5.90	0	19	0.12	–1.47

46-items	18.26	14.93	0	43	0.26	–1.48

RAN colors	68.16	22.38	33	184	1.40	4.15

RAN letters	44.29	16.33	22	117	1.50	2.91

Verbal fluency category	25.87	9.35	4	58	0.50	0.73

Verbal fluency letters	19.01	9.95	0	48	0.57	–0.25

Vocabulary	131.15	27.35	33	179	–0.69	0.39

Grammatical comprehension	11.10	4.10	2	18	–0.38	–0.90

Phonological STM	8.45	2.57	3	18	0.60	1.16

Spatial STM	9.97	2.74	2	18	–0.15	0.30

Visual STM	9.62	4.13	0	16	–0.81	–0.23

Phonological ELWM	4.88	2.01	0	10	0.15	–0.40

Visuospatial ELWM	6.97	2.97	3	17	0.96	0.74

Home literacy	44.37	5.84	27	59	–0.15	–0.12


*Note:* Abbreviations: ELWM = executive-loaded working memory, STM = short-term memory.

### Assessment

All participants were assessed in their school environment on a range of cognitive and language skills. Standardized measures were chosen where possible. All tests were administered in Swedish and the translated tests have all been used in previous research with Swedish participants. The tests for reading and language abilities had been used in a pilot study on the same population. The research group has used the cognitive tests in assessing students with ID previously. All tests were used successfully with these students. Assessments also included visual and auditory perception tests to rule out hearing and visual problems. The total testing time was estimated to be approximately 4 hours per participant, divided into sessions compliant with the school schedule. This estimation turned out to be correct. Sessions were completed during different days and breaks were allowed whenever necessary to avoid fatigue. Three test leaders (research assistant 1, months 1–14; research assistant 2, months 15–24; 1^st^ author months 1–24), who were formally trained in using all tests, conducted the assessments. All test leaders had prior experience of testing, and had been training to use the tests together. All data was recorded on paper. The data was entered by one test leader, and then re-entered by a second test leader to minimize errors. The planned test order was: word recognition, IQ, vision, phonological decoding of nonwords, hearing, visual sequential memory, reading comprehension, verbal fluency, phonological awareness, RAN, listening span, vocabulary, listening comprehension, questionnaires, digit span, grammatical understanding, odd one out span, and the Corsi blocks test. Alterations were allowed to take advantage of the whole testing session, such as moving time-consuming tests to the next session. In order to minimize the risk of fatigue or the participants experiencing feelings of failure, nearly all the tests included stopping criteria. In some cases, where stopping criteria were not a part of the original test, they were added by the research team. In studies on typically developing children, it is common to control for chronological age. However, from a developmental point of view it is more reasonable to use mental age (MA) for our sample, instead of chronological age and IQ.

### Tests

Reading comprehension was measured using the test LäSt (Elwér, Fridolfsson, Samuelsson, & Wiklund, 2016). The test consists of 17 texts of increasing length and complexity, and level of comprehension is measured via multiple-choice questions following each text. The first three texts were mandatory. After finishing these texts, testing was stopped if the participant answered less than two questions correctly. This stopping criterion was chosen on the basis that two correct answers represent more than chance. The raw score was the total number of correct answers. Listening comprehension was assessed using a subtest from Clinical Evaluation of Language Fundamentals, CELF-4 (Semel, Wiig, & Secord, 2003). The examiner orally presented three short stories and the participants were asked five questions about each story. The questions are designed to measure both understanding of different events that occurred in the story and the participant’s ability to make inferences and draw conclusions from the information provided. The raw score was the total number of correct answers.

Decoding was measured using the test LäSt (Elwér, Fridolfsson, Samuelsson, & Wiklund, 2016), where words and nonwords were read out loud from separate lists in one timed (45 seconds) and one untimed condition. Further details about this and the following tests are provided in Nilsson et al. (2021). Receptive vocabulary was measured using the Peabody Picture Vocabulary Test, Third Edition (PPVT-III) (Dunn & Dunn, 1997). Phonological awareness was measured using a test called 46-items (Olson, Forsberg, Wise, & Rack, 1994), and two subtests from the Comprehensive Test of Phonological Processing (CTOPP) (Wagner, Torgesen, & Rashotte, 1999). Grammatical understanding was measured using Test for Reception of Grammar Version 2, TROG-2 (Bishop, 2003). Verbal fluency was measured with two verbal naming tasks from the D-KEFS (Delis, Kaplan, & Kramer, 2001). Mental age was calculated using IQ and chronological age. IQ was estimated with the Vocabulary and Block Design subtests from Wechsler Intelligence Scale for Children-Fifth Edition (WISC-V) (Wechsler, 2014). Executive-loaded working memory (ELWM) was measured using odd one out span (Henry, 2001), and listening span where the participants are required to listen to a sentence spoken by the examiner, state whether it is true or false, and then retain the last word of that sentence while subsequent sentences are presented and processed. Phonological short-term memory (PSTM) was measured with forward digit span from WISC-V (Wechsler, 2014). Visuospatial short term memory (VSSTM) was measured with Visual sequential memory, a subtest taken from Test of Visual Perception Skills Revised (TVPS-R) (Gardner, 1996), and the Corsi blocks test which involves the participants mimicking the examiner who taps a sequence of up to nine identical spatially separated blocks. Home literacy was measured using questionnaires about reading habits, both for participants and parents. In addition, parents were asked about their educational level and students about their perceived reading skills. Both reading habits and educational level were scored on a four-point scale. Vision was screened with LEA-tests (Hyvärinen, Näsänen, & Laurinen, 1980), one test at a 10 feet distance and one test at a 16 inches distance. Participants with glasses were allowed to use them during testing. Hearing was screened using pure tone audiometry. For participants with hearing aids, pure tone audiometry is not applicable. However, these participants were included and coded as hearing aid users. A detailed description of the procedures for these tests is provided in Nilsson et al. (2021).

### Ethical approval

This study received ethical approval from the regional Research Ethics Committee in Linköping, Sweden (2017/139-31).

### Data analysis

All data analysis was done in R (R Core Team, 2017) and R packages. Data was analysed with structural equation modelling (SEM) with the lavaan package (Rosseel, 2012). One analysis per research question was conducted. The analysis for the first research question evaluated the support for the SVR. The SVR structure is defined with two specifications, 1) the product of listening comprehension and decoding should affect reading comprehension, and 2) no predictor variables beyond decoding, listening comprehension, and their product should influence reading comprehension directly, see ***Figure 1*** for an overview of the structures. This analysis compared the optimized SVR model with the best possible models without the two specifications above.

The analysis for the second research question evaluated two models of the delay and difference hypotheses based on the structure of the best model in the first research question. The model corresponding to the delay hypothesis was optimized with only the hypothesized predictor variables of reading and listening comprehension (grammatical skills, vocabulary, MA, and phonological executive-loaded working memory). Another model, corresponding to the difference hypothesis, was optimized without any constraint about the number of predictor variables that could be included.

Comparisons between models used an implementation of a theory of non-nested model comparison (that also handles nested comparisons) (Vuong, 1989) from the nonnest2 package (Merkle & You, 2016). If the models were distinguishable and one model had better fit than the other, that model was preferred. Otherwise, the model with least complexity (fewest paths) was preferred (the model without the product of listening comprehension and decoding together with no direct paths to reading comprehension for the first research question and the model with least predictor variables for the second research question).

Data was analysed with structural equation modelling (SEM). Models were defined based on the hypotheses, optimization of the models was carried out based on modification indices when these suggested that there should be changes to the model. Only suggestions that made sense from a theoretical point of view were implemented. Therefore, the optimization followed the rules described below, in order. As soon as a change was made to the model, the optimization was rerun starting with the first rule and this continued until no more changes were necessary. The rules were: 1) Remove non-significant paths, starting with the one with highest *p*-value; 2) Add paths to listening comprehension or decoding from the other measured variables suggested by the highest modification index; 3) Add covariance between decoding and listening comprehension if suggested by modification index; 4) Add covariance between the other measured variables suggested by the highest modification index. The fourth rule was motivated by the fact that the variables in the model are measured in a similar way, and could reflect shared measurement error.

There are no agreed upon sets of fit indices when reporting SEM results and evaluating the appropriateness of the models, but there appears to be a developing consensus to use a variety of indices. Following a combination of recommendations (Hu & Bentler, 1999; Schreiber et al., 2006), the fit of the models was evaluated using the following measures: The χ^2^, χ^2^/df, the root mean square error of approximation (*RMSEA*), the non-normed fit index (*NNFI*, also called Tucker Lewis Index, *TLI*), standardized root mean square residual (*SRMR*), and the comparative fit index (*CFI*). For χ^2^, *p* > 0.05 was used as the criterion, but because the χ^2^ statistic is sensitive to sample size, χ^2^/df < 2 (Tabachnick & Fidell, 2007) was also used. There are different suggested cut-off values for *RMSEA*, but following Hooper, Coughlan, and Mullen (2008) we chose the *RMSEA* < 0.07 criterion (Steiger, 2007). *CFI* and *NNFI* values > 0.95 (Hu & Bentler, 1999) were used as criteria for good fit. For *SRMR*, the < 0.05 criterion (Byrne, 2008) was chosen even if other researchers (Hu & Bentler, 1999) have found a more lenient criterion to be acceptable. Besides meeting all of the above criteria, all path coefficients in the model had to be significant (*p* < 0.05) for the model to be accepted. The packages papaja (Aust & Barth, 2020) and citr (Aust, 2016) were used for manuscript formatting, and tidyverse (Wickham, 2017) was used for data manipulation and the creation of plots.

## Results

Missing values were treated as missing at random, and values were imputed using the Multivariate Imputation by Chained Equations (MICE) approach, in the MICE package (van Buuren & Groothuis-Oudshoorn, 2011). The percentage of missing data was low for all variables (the maximum was 2.21 % for any variable). The decoding variable used in the SEM models was a composite of four different measures, two measures of word recognition and two measures of phonological decoding. These measures were entered into a principal component analysis (PCA) using the principal function in the psych package (Jolliffe, 2002), and the analysis favored a one-component solution. Loadings for all decoding measures ranged from 0.88 to 0.93. The proportion of explained variance for a one component solution was 81.90 %. For three assessments, composite measures were calculated by combining scores (verbal fluency 2 measures; RAN 2 measures; phonological awareness 3 measures). The sum of the z-transformed measures gave three composite variables used in the analysis. The intra-correlations between the measures ranged between 0.50 and 0.84.

Descriptive statistics of all variables before transformation are provided in ***Table 1***.

### Correlations

***Table 2*** provides correlations between all variables included in the SEM analyses. All variables except for spatial short-term memory and home literacy correlated significantly with reading comprehension. The correlation between listening comprehension and decoding was weak and non-significant (*r* = 0.10), indicating that our tests have captured two separate constructs. Furthermore, listening comprehension correlated significantly with all variables except for phonological awareness, RAN, spatial short-term memory, and home literacy. Many predictor variables also correlated significantly with each other.

**Table 2:** Correlations between all variables included in the SEM analyses.


TEST	1	2	3	4	5	6	7	8	9	10	11	12	13	14	15

1 Reading comprehension	1.00														

2 Listening comprehension	0.26	1.00													

3 Decoding	0.62	0.10	1.00												

4 Phonological awareness	0.54	0.09	0.70	1.00											

5 RAN	–0.36	–0.07	–0.58	–0.35	1.00										

6 Verbal fluency	0.31	0.25	0.29	0.27	–0.40	1.00									

7 Vocabulary	0.39	0.44	0.11	0.24	–0.11	0.32	1.00								

8 Grammatical comprehension	0.50	0.40	0.28	0.40	–0.27	0.38	0.57	1.00							

9 Phonological STM	0.35	0.20	0.44	0.49	–0.22	0.20	0.10	0.27	1.00						

10 Spatial STM	0.14	0.13	0.19	0.19	–0.25	0.18	0.11	0.23	0.23	1.00					

11 Visual STM	0.37	0.23	0.33	0.39	–0.44	0.34	0.30	0.46	0.27	0.44	1.00				

12 Phonological ELWM	0.45	0.34	0.38	0.51	–0.29	0.35	0.29	0.49	0.42	0.09	0.35	1.00			

13 Visuospatial ELWM	0.28	0.24	0.20	0.29	–0.32	0.30	0.15	0.29	0.34	0.47	0.51	0.33	1.00		

14 Home literacy	0.03	–0.13	0.00	–0.06	0.09	–0.04	0.03	–0.17	0.01	–0.14	–0.06	–0.02	–0.12	1.00	

15 Mental age	0.44	0.27	0.27	0.31	–0.37	0.30	0.44	0.43	0.22	0.25	0.43	0.25	0.44	0.03	1.00


### Structural equation modeling

**Figure 3:**
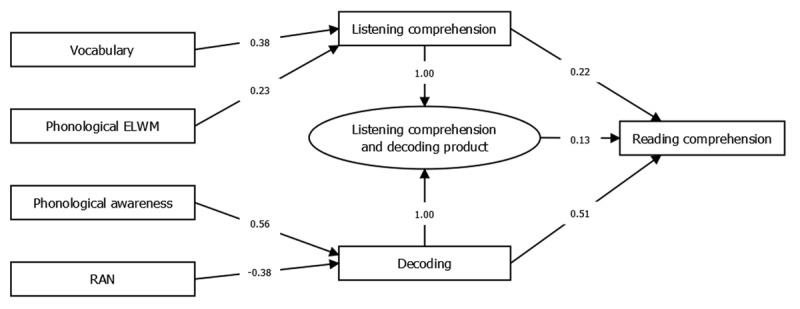
Path model showing the optimized model for hypothesis 1a, the Simple View of Reading hypothesis.

***Figure 3*** shows a plot of the optimized model for hypothesis 1a, that the Simple View of Reading is applicable. This model was optimized with the following constraints: 1) the product of decoding and listening comprehension should influence reading comprehension, 2) no predictor variables beyond decoding, listening comprehension, and their product should influence reading comprehension directly. This model did not provide a good fit to the data on any of the fit indices, χ^2^(16) = 333.99, *p* = < .001, χ^2^/df = 20.87, *RMSEA* = 0.38 (*CI*_90%_ [0.35, 0.42]), *NNFI* = 0.11, *SRMR* = 0.24, *CFI* = 0.49. All paths were significant (*p* < 0.05), except for the path from the product of decoding and listening comprehension to reading comprehension (*p* = .051).

**Figure 4:**
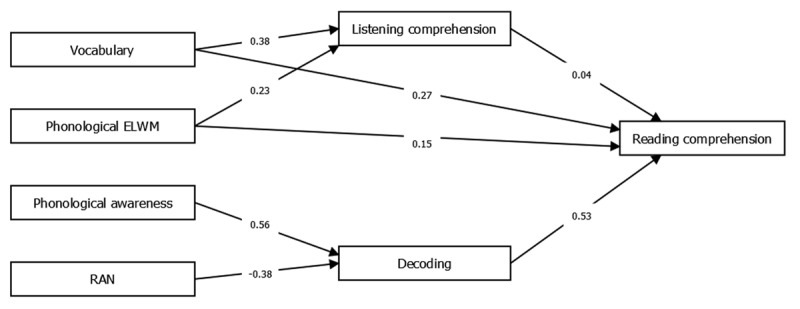
Path model showing the optimized model for hypothesis 1b, Simple View of Reading is not applicable.

***Figure 4*** shows a plot of the optimized model for hypothesis 1b, that the Simple View of Reading is not applicable. This model was set up without the constraints listed for hypothesis 1a, meaning that the product of decoding and listening comprehension did not have to influence reading comprehension, and all predictors were allowed to influence reading comprehension directly. The model provided a good fit to the data on all fit indices, χ^2^(8) = 8.81, *p* = .390, χ^2^/df = 1.10, *RMSEA* = 0.02 (*CI*_90%_ [0, 0.11]), *NNFI* = 0.99, *SRMR* = 0.03, *CFI* = 1.00. All paths were significant (*p* < 0.05), except for the path from listening comprehension to reading comprehension (*p* = .532). Unstandardized and standardized estimates for all paths in the model of hypothesis 1b can be found in ***Table 3***.

**Table 3:** Unstandardized and standardized estimates for all paths in the optimized model for hypothesis 1b, the Simple View of Reading is not applicable.


			ESTIMATES		

LEFT TERM	OPERATOR	RIGHT TERM	UNSTANDARDIZED	STANDARDIZED	*p*

Regressions					

Reading comprehension	<-	Decoding	6.63	0.53	<.001

Reading comprehension	<-	Listening comprehension	0.14	0.04	.532

Reading comprehension	<-	Phonological ELWM	0.95	0.15	.028

Reading comprehension	<-	Vocabulary	0.12	0.27	<.001

Listening comprehension	<-	Phonological ELWM	0.42	0.23	.003

Listening comprehension	<-	Vocabulary	0.05	0.38	<.001

Decoding	<-	Phonological awareness	0.22	0.56	<.001

Decoding	<-	RAN	–0.21	–0.38	<.001

Covariances					

Vocabulary	<–>	Phonological awareness	14.59	0.21	.012

Vocabulary	<–>	Phonological ELWM	13.99	0.26	.003

Phonological ELWM	<–>	Phonological awareness	2.56	0.50	<.001

Phonological ELWM	<–>	RAN	–0.97	–0.27	.002

RAN	<–>	Phonological awareness	–1.54	–0.33	<.001

Reading comprehension	<–>	Reading comprehension	76.12	0.49	<.001

Decoding	<–>	Decoding	0.39	0.39	<.001

Listening comprehension	<–>	Listening comprehension	10.43	0.76	<.001

Vocabulary	<–>	Vocabulary	742.27	1.00	<.001

Phonological ELWM	<–>	Phonological ELWM	3.94	1.00	<.001

Phonological awareness	<–>	Phonological awareness	6.54	1.00	<.001

RAN	<–>	RAN	3.36	1.00	<.001


A comparison between the two models was made using an implementation of a theory of non-nested model comparison. This showed that the models were distinguishable (*ω*^2^ = 0.11, *p* = .014), and that model 1b had the best fit (*z* = 2.82, *p* = .002). In other words, the hypothesis that the Simple View of Reading is not applicable was supported.

To answer the second research question, models corresponding to hypothesis 2a (delay) and hypothesis 2b (difference) were set up. As described earlier, these models were based on the best model from the first research question, i.e. model 1b, specified according to the hypotheses and optimized with the same rules as for the first analysis. The optimized models for both the delay hypothesis and the difference hypothesis were identical to each other and identical to model 1b. Unsurprisingly, the comparison between the two identical models showed that they were indistinguishable. This means that it was not possible to choose a delay or difference hypothesis based on the comparison. However, as vocabulary and phonological ELWM were the only significant predictors of listening comprehension and reading comprehension in the final model, the delay hypothesis could be regarded as partly supported.

## Discussion

The first aim of the present study was to examine the applicability of the Simple View of Reading (SVR) in a sample of adolescents with non-specific ID. The second aim was to identify the concurrent cognitive and linguistic predictors of listening and reading comprehension in the same sample to evaluate whether delay or difference approaches best accounted for the findings. The results showed that the SVR was not supported in this group. For the second aim, our results were for the most part consistent with a delayed profile of predictors associated with listening and reading comprehension.

The results from the present study show that listening comprehension and decoding alone are not sufficient when explaining reading comprehension abilities in adolescents with non-specific ID. In addition to these components, reading comprehension appeared to be influenced by vocabulary and phonological executive-loaded working memory (ELWM). In fact, when direct paths were established from vocabulary and phonological ELWM to reading comprehension, the impact of listening comprehension decreased to almost zero. These results are to some extent in line with results from Ouellette and Beers (2010), where the authors argue for a “not so simple view of reading.” In their study, which investigated the SVR in a sample of typically developing children, vocabulary was found to predict reading comprehension even after decoding, listening comprehension, irregular word recognition, and phonological awareness were accounted for. Furthermore, vocabulary accounted for a higher percentage of variance compared to listening comprehension (Ouellette & Beers, 2010). Another study found that vocabulary contributed significantly to reading comprehension in typically developing children even when decoding and listening comprehension were accounted for (Tunmer & Chapman, 2012). Studies of variables associated with reading comprehension in individuals with ID are sparse, but Nash and Heath (2011) found an association between vocabulary and reading comprehension in a sample with Down Syndrome.

The finding of the present study that phonological ELWM, rather than phonological STM, predicts reading comprehension suggests that the information processing component of ELWM might be the crucial component of memory associated with reading comprehension. However, listening span is a verbally mediated assessment of working memory, and it has been suggested that these kind of measures are heavily dependent on vocabulary and other verbal abilities (Nation, Adams, Bowyer-Crane, & Snowling, 1999; Stothard & Hulme, 1992). For example, Nation, Adams, Bowyer-Crane, and Snowling (1999) argued that the reason that their sample of poor comprehenders exhibited difficulties with listening span was due to the nature of the assessment, where the participant is required to listen to a sentence, state whether it is true or not, and then retain the last word of the sentence. Since poor comprehenders are, by definition, less skilled at comprehending sentences compared to typical readers this could explain the difficulties, and the same line of reasoning could be applied to the sample with ID in the current study. In contrast, a study by Cain, Oakhill, and Bryant (2004) showed that phonological ELWM explained unique variance in reading comprehension in a sample of typically developing children, even after accounting for word reading and verbal ability. A similar pattern emerged in a study investigating the predictors of listening comprehension (Kim, 2016). Phonological ELWM was found to directly predict listening comprehension, over and above vocabulary and grammatical knowledge. This could imply that there are non-language processes, such as the ability to process multiple sources of concurrent information at the same time, that are of importance for reading comprehension.

In our adolescent readers, decoding had a strong and significant impact on reading comprehension, while the contribution of listening comprehension remained weak and non-significant. Some studies have shown that the relative contribution of the components in the SVR shifts over time (Juel, Griffith, & Gough, 1986; Lervåg, Hulme, & Melby-Lervåg, 2017; Verhoeven & van Leeuwe, 2012). In the early stages of reading development, decoding has a strong impact on reading comprehension. Once decoding is mastered, its importance for reading comprehension decreases while the importance of listening comprehension increases. The results of our study indicate that the readers with non-specific ID may not have reached the level of decoding skills required to go through this developmental shift. Unlike our analyses, Roch and Levorato (2009) found support for the listening comprehension component of the SVR, when applying the framework to a sample consisting of 23 individuals with Down Syndrome aged between 11 and 18 years. Their study involved two regression analyses, where listening comprehension and two separate measures of word decoding (fluency and accuracy) were entered. Listening comprehension significantly explained 19.2% and 16.9%, respectively, of the variance in reading comprehension, while none of the decoding measures accounted for unique variance (Roch & Levorato, 2009). However, this study did not include other possible predictors of reading comprehension, and this may have made the role of listening comprehension more prominent.

When evaluating the delay and difference hypotheses, the models corresponding to our stated hypotheses were found to be identical, preventing the non-nested model comparison to favor either the delay or difference model. However, the predictors that emerged in the identical models (vocabulary and phonological ELWM) partly corresponded to the hypothesized predictors in the delay hypothesis. This indicates that the variables explaining listening comprehension and reading comprehension in a sample with non-specific ID were similar to the variables found in research on typically developing children (e.g. Braze, Tabor, Shankweiler, & Mencl, 2007; Kim, 2015, 2016; Muter, Hulme, Snowling, & Stevenson, 2004; Ouellette & Beers, 2010).

Based on the operationalization of the concepts in the current study, our findings suggest that vocabulary and phonological ELWM are of direct importance to reading comprehension, rather than their influence being entirely mediated through listening comprehension. For both vocabulary and phonological ELWM, there was a direct link to reading comprehension and an indirect link to reading comprehension via listening comprehension. However, it should be acknowledged that these findings are not as problematic for the SVR as some alternative findings could have been. For example, finding relationships to reading comprehension that involved variables unrelated to listening comprehension or decoding, such as visuospatial working memory, would have been even less consistent with the SVR. A related issue is that other theories about reading comprehension, such as the Lexical Quality Hypothesis (Perfetti, 2007) and the Reading Systems Framework (Perfetti & Stafura, 2014), claim that lexical knowledge can add to the explanation of reading comprehension. Our findings suggest that future research should investigate these mechanisms.

### Limitations

The present study aimed to include 150 participants and achieved a final sample size of 136 which was closed to the desired target. The achieved sample size was considered large enough to proceed with our planned analyses.

It is also important to consider the fact that adolescents who had non-specific ID and other co-occurring conditions such as ASD or ADHD were included in the current study. On the one hand, this decreased the internal validity and made it more difficult to draw firm conclusions about the impact of having a non-specific ID, as there may be effects of having both ASD/ADHD and ID that we were unable to unpick. On the other hand, this increased the external validity as our sample reflects the population we are trying to understand.

## Data accessibility statement

Raw data with guidance notes, analysis script, stage 1 IPA manuscript, and a laboratory log are available at: *https://osf.io/6wfhb/*
